# Orbscan IIz multimodal corneal topography dataset of anterior axial power maps with structured clinical parameters for unsupervised and self-supervised representation learning

**DOI:** 10.1016/j.dib.2026.113138

**Published:** 2026-08-10

**Authors:** Ali Mustafa Ali Alshaykha, Qabas A. Hameed, Mohammed Basim Omar

**Affiliations:** a College of Basic Education, Al-Shirqat, University of Tikrit, Tikrit, Iraq; b College of Computer Science and Mathematics, University of Tikrit, Tikrit, Iraq

**Keywords:** Artificial intelligence, Optical character recognition, Image preprocessing, Ophthalmic imaging, Machine learning, Open research data

## Abstract

We present a multimodal corneal topography dataset comprising 3000 anterior axial power maps acquired from 3000 unique patients using the Orbscan IIz slit-scanning device (Bausch & Lomb) at a single private ophthalmology clinic in Iraq. The examinations were performed during routine clinical practice between 2007 and 2025 using the same diagnostic device throughout the acquisition period. Each image is paired with 19 structured clinical parameter fields extracted from the original device output using an automated OCR-based pipeline. These parameters include simulated keratometry astigmatism (SimK_Astig), maximum and minimum corneal power (MaxK and MinK), zone-specific irregularity indices, mean power, astigmatic power, steep and flat axis orientations across 3 mm and 5 mm optical apertures, corneal pachymetry at the thinnest point with spatial coordinates, anterior chamber depth, white-to-white diameter, pupil diameter, and angle kappa with intercept coordinates. All images were fully anonymized, cropped to the central map region, resized to 1024 × 1024 pixels, and saved in lossless PNG format. Following a quality control pipeline assessing image integrity, metadata completeness, and anatomical plausibility, 2633 image–metadata pairs satisfied the predefined inclusion criteria and constitute the quality-controlled subset characterized in this article. The dataset is released without diagnostic labels by design, positioning it as a foundation for unsupervised, self-supervised, and semi-supervised representation learning in anterior segment analysis. The complete dataset is available on Mendeley Data (DOI: 10.17,632/78wt2nc387.3) under a CC BY-NC 4.0 license.

Specifications Table

**Table:** 

Subject	Computer Science; Ophthalmology; Medical Imaging; Artificial Intelligence
Specific subject area	Multimodal corneal topography imaging dataset for anterior segment analysis, unsupervised learning, self-supervised representation learning, and multimodal medical AI research
Type of data	Image [PNG] Table [CSV] Text [README]
Data collection	Anterior axial power maps were exported from a Zyoptix Diagnostic Workstation (Orbscan IIz, Bausch & Lomb; Serial No ZDW-08/02–0015) during routine clinical examinations. Clinical parameters were extracted from device-generated report regions using Tesseract OCR (v5) followed by rule-based parsing and post-processing. A quality control pipeline assessed image integrity, metadata completeness, and anatomical plausibility. Inclusion criterion: one image per unique patient; no repeat visits or bilateral paired images were included.
Data source location	Private ophthalmology clinic, Iraq Acquisition period: 2007–2025
Data accessibility	Repository name: Mendeley Data Data identification number: 10.17632/78wt2nc387.2 [20] Direct URL to data: https://doi.org/10.17632/78wt2nc387.2 License: CC BY-NC 4.0
Related research article	Not applicable

## 1 Value of the Data

• To our knowledge, this dataset represents the first openly available multimodal corneal topography resource based on the Orbscan IIz slit-scanning platform. It provides paired anterior axial power maps and structured clinical parameters, addressing the scarcity of publicly accessible, platform-specific anterior segment imaging datasets for artificial intelligence research [[1], [2], [3]].
• The dataset is valuable for researchers in medical image analysis, ophthalmic artificial intelligence, and representation learning who require real-world, unlabeled multimodal data for developing unsupervised, self-supervised, and semi-supervised learning algorithms [2,4,5]. Its paired image–metadata structure reflects the actual output of clinical corneal topography examinations, making it suitable for clinically grounded AI model development.
• The structured metadata include clinically routine parameters that are rarely available together in open datasets, such as angle kappa with intercept coordinates, zone-specific irregularity indices, mean power, astigmatic power across 3 mm and 5 mm optical apertures, and steep and flat axis orientations [6,7]. These parameters are relevant to refractive surgery screening, ectasia risk assessment, intraocular lens planning, and anterior segment analysis [8].
• The absence of diagnostic labels is intentional and expands the range of possible research applications [2,4]. Researchers can use the dataset for clustering, contrastive image–metadata pretraining, anomaly detection, and future researcher-defined annotation protocols [[12], [19]]. Partial annotation of selected subsets may further enable supervised benchmarking while preserving the unlabeled majority for representation learning [[9], [10], [11]].

## 2 Background

Corneal topography provides spatially dense maps of the anterior corneal surface, encoding curvature, refractive power, and irregularity information that is essential for clinical decision-making in refractive surgery screening, ectasia risk stratification, and contact lens fitting [1,12]. The Orbscan IIz is a slit-scanning topography system that acquires anterior and posterior corneal surface data simultaneously and generates a structured clinical summary report alongside the topographic map, offering a richer data output than Placido-disc-based systems that produce only curvature-derived maps [12,13].

Despite the clinical ubiquity of corneal topography imaging, open datasets in this domain remain scarce, particularly those pairing topographic images with structured numerical clinical parameters. Existing publicly available resources are predominantly diagnostic — built around labeled keratoconus versus normal classifications — and rely on Pentacam tomography rather than slit-scanning topography [9,2,10,11]. No comparable open resource exists for the Orbscan IIz platform. This gap limits the development of platform-specific AI models and precludes unsupervised exploration of the natural structure embedded in large-scale, unlabeled topographic archives.

The present dataset was compiled from a retrospective archive of routine clinical examinations performed at a single private ophthalmology clinic in Iraq between 2007 and 2025. It was assembled to provide the research community with a large-scale, multimodal, and unlabeled corneal topography resource that supports unsupervised, self-supervised, and semi-supervised learning paradigms in anterior segment analysis.

## 3 Data Description

The released dataset is hosted on Mendeley Data (DOI: 10.17,632/78wt2nc387.3) under a CC BY-NC 4.0 license. The repository contains an image archive comprising 3000 processed PNG files (Images.zip), one structured metadata file (metadata.csv), a plain-text documentation file (README.txt), a Python analysis script (analysis_script.py) that reproduces all descriptive statistics and figures reported in this article, and an analysis-ready numeric CSV file (metadata_numeric.csv) in which composite text fields have been parsed into separate numeric columns and anatomical plausibility filtering has been applied. The README.txt covers the complete repository structure, field-level data dictionary, Python loading examples, and step-by-step instructions for reproducing the quality-controlled subset and all reported results. The dataset was organized to enable direct linkage between each anterior axial power map and its corresponding structured clinical parameters.

Repository file structure:

Images.zip - 3000 anonymized PNG images (1024 × 1024 px, lossless)

metadata.csv - Original OCR-extracted metadata (3000 records × 20 columns)

metadata_with_QC.csv - Adds QC_status column: PASS (N = 2633) or FAIL (N = 367)

metadata_numeric.csv - Composite fields parsed into 30 numeric columns

analysis_script.py - Reproduces all tables and figures in this article

requirements.txt - Python package dependencies

README.txt - Comprehensive documentation

LICENSE.txt - CC BY-NC 4.0 license

Table 1 summarizes key characteristics of related publicly available corneal topography datasets. To our knowledge, no comparable open resource combines anterior axial power maps with the full set of Orbscan IIz clinical summary parameters while remaining accessible for unsupervised research paradigms (Table 2).

**Table 1:** Comparison of selected publicly available corneal topography datasets.

Dataset	Device	Images (N)	Labels	Image Type	Metadata
Ghorbani et al. [9]	Pentacam	6000	Yes (6 classes)	4 map types	No tabular parameters
Lazouni et al. [14]	Orbscan II	∼200	Yes (KC/Normal)	Anterior maps	Image-derived descriptors
Agharezaei et al. [10]	Unspecified	1758	Yes (KC/Normal)	Topographic maps	No structured metadata
Al-Timemy et al. [11]	Pentacam	∼300	Yes (KC/Normal)	4 map types	Pentacam indices only
Zéboulon et al. [2]	Unspecified	7019	Auto (3 groups)	Topographic maps	No released dataset
**Present dataset**	**Orbscan IIz**	**3000**	**No (unlabeled)**	**Anterior axial power**	**19 structured fields**

**Table 2:** Column descriptions, data formats, and example values for metadata.csv.

Column Name	Description	Format	Example Value
**filename**	Image identifier	Text	P0001_AxialPower_Anterior
**SimK_Astig**	Keratometric astigmatism magnitude	Text (D @ deg)	−2.4 D @ 3 deg
**MaxK**	Maximum corneal power	Text (D @ deg)	47.1 D @ 93 deg
**MinK**	Minimum corneal power	Text (D @ deg)	44.7 D @ 3 deg
**Zone3mm_Irreg**	3 mm zone irregularity index	Text (± D)	± 1.1 D
**Zone3mm_MeanPwr**	3 mm zone mean power	Text (D ± D)	50.9 ± 0.6 D
**Zone3mm_AstigPwr**	3 mm zone astigmatic power	Text (D ± D)	2.2 ± 0.9 D
**Zone3mm_SteepAxis**	3 mm steep axis orientation	Text (deg ± deg)	92 ± 10 deg
**Zone3mm_FlatAxis**	3 mm flat axis orientation	Text (deg ± deg)	1 ± 10 deg
**Zone5mm_Irreg**	5 mm zone irregularity index	Text (± D)	± 2.0 D
**Zone5mm_MeanPwr**	5 mm zone mean power	Text (D ± D)	50.4 ± 0.9 D
**Zone5mm_AstigPwr**	5 mm zone astigmatic power	Text (D ± D)	2.0 ± 1.8 D
**Zone5mm_SteepAxis**	5 mm steep axis orientation	Text (deg ± deg)	91 ± 24 deg
**Zone5mm_FlatAxis**	5 mm flat axis orientation	Text (deg ± deg)	1 ± 23 deg
**WhiteToWhite_mm**	White-to-white corneal diameter	Numeric (mm)	11.4
**PupilDiameter_mm**	Pupil diameter	Numeric (mm)	3.8
**Thinnest**	Thinnest corneal point with coordinates	Text (µm @ x, y)	531 µm @ (0.8, −0.2)
**ACD_Ep_mm**	Anterior chamber depth (epithelium)	Numeric (mm)	3.62
**Kappa**	Angle kappa	Text (deg @ deg)	6.86 deg @ 337.29 deg
**KappaIntercept**	Kappa intercept coordinates	Text (x, y)	0.37, 0.01

File Structure

Images/

The image directory contains 3000 PNG files, each representing an anterior axial power map acquired from a unique patient. Image files follow the naming convention PXXXX_AxialPower_Anterior.png, where XXXX is a zero-padded anonymized identifier (e.g., P0001_AxialPower_Anterior.png).

metadata.csv

The metadata file contains 3000 records and 20 columns (one image identifier and 19 structured clinical parameter fields). Each row corresponds to one unique patient and is linked to its corresponding image through the filename field.

### 3.1 Image–metadata linkage

Records are linked through the filename field in metadata.csv. The value corresponds directly to the PNG filename without the file extension. Image filenames follow the format PXXXX_AxialPower_Anterior.png, where XXXX is a sequential zero-padded integer assigned during dataset construction. These identifiers are arbitrary and carry no demographic, clinical, or temporal meaning; no patient identifiers can be recovered from them. Note: the original raw Orbscan IIz device screenshots are not included in the released dataset because the embedded device interface elements contain patient-identifiable information that cannot be fully separated from the underlying image data. Only the anonymized, cropped, and standardized PNG images are released (e.g., P0001_AxialPower_Anterior → P0001_AxialPower_Anterior.png).

### 3.2 Quality-controlled subset

Of the 3000 image–metadata pairs in the released dataset, 2633 satisfied the predefined quality control, metadata completeness, and anatomical plausibility criteria and were used for the descriptive statistical characterization reported in this article. The remaining 367 records are retained in the released dataset but may contain incomplete OCR-derived metadata fields. To facilitate reproducibility, a companion file metadata_with_QC.csv — which extends the original metadata.csv by adding a single QC_status column — has been released, where records satisfying all criteria are labelled PASS (N = 2633) and records failing one or more criteria are labelled FAIL (N = 367). Users wishing to reproduce the descriptive statistics reported in this article should filter for QC_status = PASS prior to analysis.

### 3.3 Missing values

Missing values are present in both metadata.csv and metadata_with_QC.csv, as these files share the same raw OCR-extracted values. The highest missing value counts in the full released dataset (N = 3000) are: MaxK (233; 7.8%), MinK (97; 3.2%), and SimK_Astig (67; 2.2%). In metadata_numeric.csv, additional values are set to NaN where the extracted numeric value fell outside the predefined anatomical plausibility range. Missing values are represented as empty fields in all CSV files.

File selection guidance: use metadata.csv for access to raw OCR-extracted values; use metadata_with_QC.csv and filter QC_status = PASS to reproduce the quality-controlled subset (N = 2633) and all descriptive statistics reported in this article; use metadata_numeric.csv for computational analyses requiring separate numeric columns with plausibility filtering applied.

*Example record (P0001_AxialPower_Anterior): filename = P0001_AxialPower_Anterior; corresponding image = P0001_AxialPower_Anterior.png; SimK_Astig = −2.4 D @ 3* *deg; MaxK = 47.1 D @ 93* *deg; MinK = 44.7 D @ 3* *deg; Zone3mm_Irreg = ± 1.1 D; Thinnest = 531* *µm @ (0.8, −0.2); ACD_Ep_mm = 3.62; Kappa = 6.86* *deg @ 337.29* *deg; QC_status = PASS.*

## 4 Experimental Design, Materials and Methods

### 4.1 Dataset acquisition

Anterior axial power map images were acquired from 3000 unique patients during routine clinical examinations at a private ophthalmology clinic in Iraq between 2007 and 2025. All examinations were performed using a single Zyoptix Diagnostic Workstation (Orbscan IIz, Bausch & Lomb; Serial No ZDW-08/02–0015) throughout the acquisition period. One image was included per patient; no bilateral paired images or repeat examinations from the same patient were incorporated into the released dataset. The dataset was collected retrospectively from archived clinical examinations; no examinations were performed specifically for research purposes.

### 4.2 Image preprocessing

Raw device screenshots were exported from the Orbscan IIz system as BMP files. An automated region of interest (ROI) cropping procedure was applied using fixed pixel coordinates (x1=345, y1=70, x2=1628, y2=1293) uniformly across all 3000 images without exception; no manual correction was required for any image. The crop isolates the central topographic map and removes surrounding device interface elements including the software toolbar, patient identification fields, and device header. Pixel regions containing patient-identifiable information were permanently removed before release. The cropped images were resized to a standardized 1024 × 1024 pixel matrix using Lanczos resampling (PIL.Image.LANCZOS) and saved in lossless PNG format to avoid compression artifacts.

### 4.3 OCR-Based metadata extraction

Extraction of the 19 structured clinical parameter fields required a dedicated computational pipeline because the text embedded within Orbscan IIz screenshots is not directly accessible to standard text parsers. The pipeline was implemented using Tesseract OCR v5 (–oem 3 –psm 6, lang=eng) and proceeded through four sequential steps:

1. The clinical summary table region was isolated from each screenshot using predefined ROI coordinates.
2. The isolated region was converted to high-contrast grayscale and upscaled by a factor of 2× to improve character recognition accuracy.
3. Tesseract OCR (v5) was applied to extract raw text strings.
4. Rule-based regular expressions were applied to identify and extract target clinical parameter values.

A targeted post-processing step addressed OCR-related decimal point dropouts. Extracted values were evaluated against predefined anatomical plausibility ranges, and decimal points were reinserted only when the raw OCR output lacked a decimal separator and the corrected value fell within the predefined plausibility range. Common corrected examples include: 471 → 47.1 D (MaxK); 24 → 2.4 D (SimK_Astig); 114 → 11.4 mm (WhiteToWhite); 362 → 3.62 mm (ACD_Ep). The final extracted values were compiled into the master metadata.csv file.

### 4.4 Quality control and dataset validation

A two-stage quality control pipeline was applied. In the first stage, image integrity was assessed to identify files with severe blur, major misalignment, corrupted file headers, or incomplete corneal map capture. In the second stage, metadata completeness and plausibility were assessed by calculating missing value ratios across all extracted fields and flagging values that were missing, implausible, or unrecoverable after post-processing.

Following both stages, 2633 image–metadata pairs satisfied the predefined criteria and constitute the quality-controlled subset. The remaining 367 records are retained without imputation. A randomly selected subset of **150 finalized records** (6.3%) was manually cross-referenced against original device screenshots to verify filename linkage and OCR-derived metadata consistency.

### 4.5 Descriptive statistical analysis

**Fig. 1:**
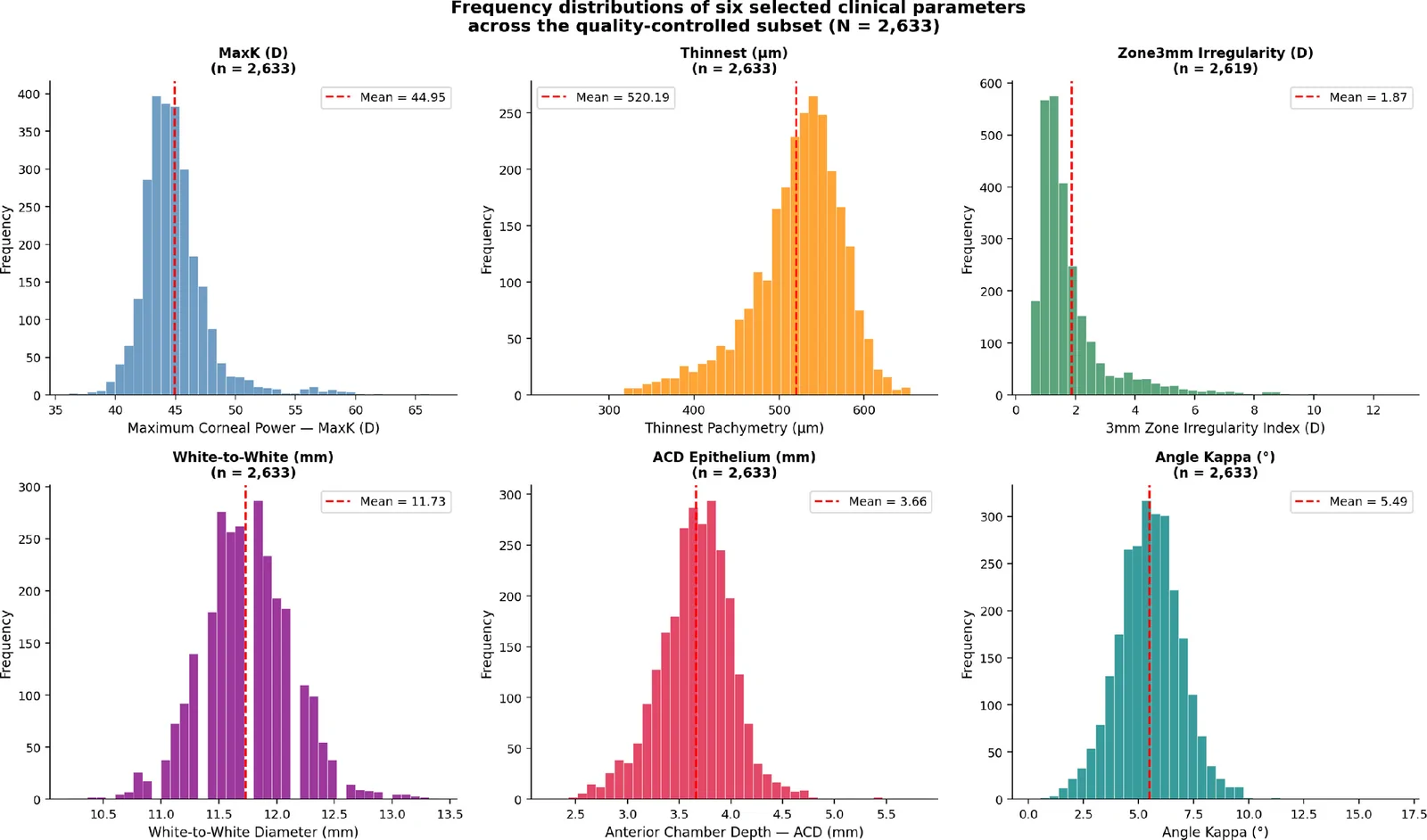
Frequency distributions of six selected clinical parameters across the quality-controlled subset. Sample sizes (n) shown per panel reflect records with valid values after plausibility filtering. Red dashed lines indicate distribution means. Right-skewed distributions in MaxK and Zone3mm_Irreg demonstrate the presence of high-curvature and irregularity extremes within the dataset.

**Fig. 2:**
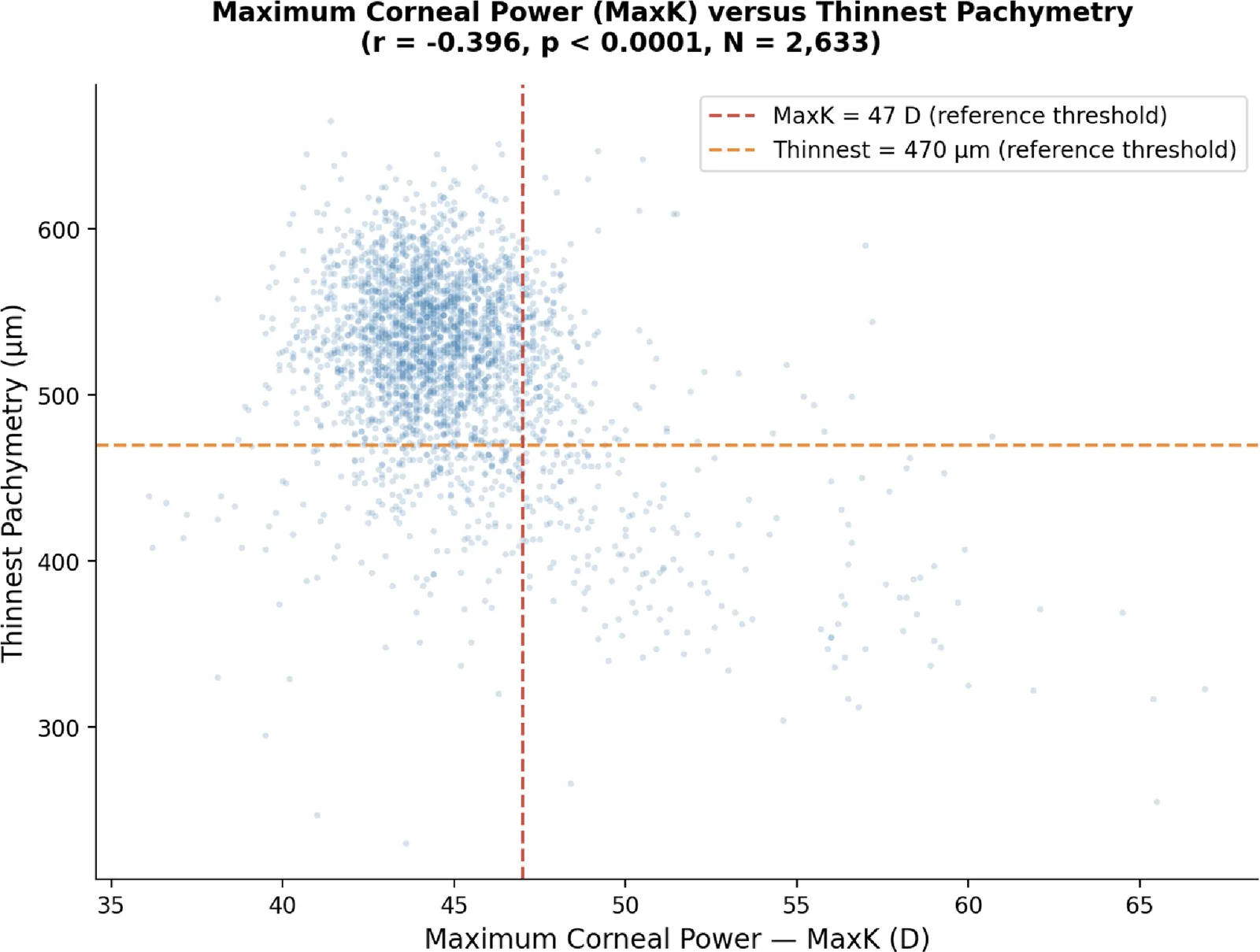
Bivariate scatter plot of Maximum Corneal Power (MaxK) versus Thinnest Pachymetry across the quality-controlled subset (N = 2633 valid pairs). The inverse relationship (r = −0.396, p < 0.0001) demonstrates the expected inverse association between higher corneal power and reduced pachymetry. Reference thresholds for MaxK = 47 D and Thinnest = 470 µm are indicated by dashed lines.

Descriptive statistics were computed for the 2633 quality-controlled records. For composite text fields, the primary numeric value was extracted prior to analysis. For SimK_Astig, the absolute magnitude was used. For KappaIntercept, the Euclidean distance from origin was computed. All extracted values were evaluated against clinical plausibility ranges before statistical analysis; values outside these ranges were treated as missing and are documented in Table 3. For each parameter: mean, standard deviation (SD), median, minimum, maximum, Q1, Q3, IQR, skewness, excess kurtosis, outlier rate, and Shapiro–Wilk (SW) normality test were calculated. Pearson correlation coefficients examined bivariate relationships between selected parameters (Fig. 2). All analyses were performed on the quality-controlled subset using metadata_numeric.csv for numeric extraction. The complete analysis pipeline was implemented in Python 3.9 or later using the following libraries: pandas (≥ 2.0), NumPy (≥ 1.26), scipy.stats (≥ 1.11), and matplotlib (≥ 3.8). The OCR extraction pipeline used OpenCV (cv2 ≥ 4.8), Pillow (≥ 10.0), and Tesseract OCR 5.x. Exact version requirements are provided in requirements.txt deposited on Mendeley Data (Fig. 1).

**Table 3:** Descriptive statistics of the 19 clinical parameters across the quality-controlled subset (N up to 2633; individual N varies due to missing values after plausibility filtering). SW: Shapiro–Wilk normality test.

Parameter	N	Mean	SD	Median	Min	Max	Q1	Q3	IQR	Skewness	Kurtosis	Outliers (%)	SW p
SimK_Astig (D, absolute)	2631	1.87	1.58	1.4	0.0	13.0	0.8	2.5	1.7	2.154	7.283	4.1	<0.0001
MaxK (D)	2633	44.95	2.95	44.5	36.1	66.9	43.3	46.0	2.7	2.114	8.833	5.2	<0.0001
MinK (D)	2633	43.07	2.24	42.9	35.1	59.5	41.8	44.2	2.4	1.113	5.283	3.3	<0.0001
Zone3mm_Irreg (D)	2350	2.61	2.47	1.5	0.7	12.9	1.2	2.4	1.2	1.842	1.974	16.1	<0.0001
Zone3mm_MeanPwr (D)	1838	49.21	2.52	48.7	41.0	63.3	47.7	50.7	3.0	1.03	3.303	3.2	<0.0001
Zone3mm_AstigPwr (D)	2572	1.82	1.37	1.4	0.0	12.1	0.8	2.5	1.7	1.727	5.066	3.3	<0.0001
Zone3mm_SteepAxis (°)	2617	91.54	31.65	92.0	0.0	180.0	81.0	105.0	24.0	−0.213	1.352	14.7	<0.0001
Zone3mm_FlatAxis (°)	2592	83.66	71.29	67.0	1.0	180.0	12.0	167.0	155.0	0.188	−1.714	0.0	<0.0001
Zone5mm_Irreg (D)	2390	2.25	1.62	1.7	0.7	13.3	1.4	2.3	0.9	3.184	11.69	9.8	<0.0001
Zone5mm_MeanPwr (D)	1820	48.56	2.09	48.2	41.7	60.6	47.3	50.1	2.8	0.672	2.098	1.5	<0.0001
Zone5mm_AstigPwr (D)	2608	1.66	1.18	1.4	0.1	15.0	0.8	2.3	1.5	1.857	9.323	2.0	<0.0001
Zone5mm_SteepAxis (°)	2619	92.3	30.54	92.0	0.0	180.0	81.0	104.0	23.0	−0.119	1.699	14.3	<0.0001
Zone5mm_FlatAxis (°)	2572	83.94	71.6	63.0	1.0	180.0	13.0	166.0	153.0	0.181	−1.75	0.0	<0.0001
WhiteToWhite (mm)	2633	11.73	0.4	11.7	10.2	13.4	11.5	12.0	0.5	0.172	0.748	1.9	<0.0001
PupilDiameter (mm)	2633	4.42	0.81	4.4	1.7	8.4	3.9	4.9	1.0	0.721	1.802	1.8	<0.0001
Thinnest (µm)	2633	520.19	56.01	528.0	230.0	665.0	493.0	558.0	65.0	−1.006	1.696	3.9	<0.0001
ACD_Ep (mm)	2633	3.66	0.36	3.68	2.25	5.82	3.45	3.89	0.44	−0.018	1.648	2.5	<0.0001
Kappa (°)	2633	5.49	1.48	5.49	0.11	16.88	4.53	6.4	1.87	0.332	2.345	1.6	<0.0001
KappaIntercept (mm)	2630	0.55	0.28	0.52	0.0	2.38	0.35	0.71	0.36	0.774	1.524	1.6	<0.0001

## Limitations

Several limitations should be considered when using this dataset.

**First**, all images were acquired at a single private ophthalmology clinic in Iraq using a single Orbscan IIz device (Serial No ZDW-08/02–0015) over an 18-year period (2007–2025). While this design ensures internal consistency and eliminates inter-device variability, it introduces important constraints on generalizability. The dataset does not represent the diversity of patient populations, geographic regions, or clinical settings encountered in real-world deployment. Models trained exclusively on this dataset may not generalize to other Orbscan IIz units, to slit-scanning devices from different manufacturers, or to Placido-disc or Scheimpflug-based topography systems. Researchers developing AI models for broad clinical use are strongly encouraged to supplement this dataset with data from multiple devices, institutions, and populations [15,16].

**Second**, the dataset does not include diagnostic labels, clinical diagnoses, or patient demographic information [[17], [18]]. The absence of labels is intentional and supports unsupervised research paradigms but prevents direct supervised use without additional annotation [[9], [10], [11]].

**Third**, the dataset provides a single cross-sectional image per patient with no longitudinal follow-up, limiting applicability for temporal or disease progression modeling.

**Fourth**, clinical metadata values were extracted via an automated OCR-based pipeline. Although rule-based post-processing and anatomical plausibility filtering were applied, residual extraction errors cannot be fully excluded.

**Fifth**, missing values are present across several metadata fields (highest rate: MaxK, 7.8%). Records were retained without imputation to preserve transparency.

**Sixth**, metadata fields preserve composite text strings reflecting the original Orbscan IIz report format. Numerical analyses require prior parsing to isolate primary scalar values, as demonstrated in Section 4.5.

*Despite these limitations, the dataset remains a useful multimodal resource for anterior segment image analysis, representation learning, anomaly detection, clustering, and future researcher-defined annotation protocols.*

## Ethics Statement

The dataset was compiled retrospectively from anonymized Orbscan IIz corneal topography images acquired during routine clinical examinations between 2007 and 2025. No new patient examinations were conducted for the purpose of this study. All patient-identifiable information, including patient names, registration numbers, and examination dates, was permanently removed before dataset preparation, analysis, and release. The released dataset does not contain patient names, personal identifiers, demographic information, or diagnostic labels.

Written authorization for data access, processing, anonymization, and research publication was obtained from the treating physician and original data custodian, Dr. Abdullah Ahmed Al-Juboori, Specialist Ophthalmologist (Iraqi Medical Association Registration No 26,750). The data custodian confirmed that the original examinations were collected as part of routine clinical care and that the released dataset had been de-identified prior to publication in accordance with accepted research data protection practices. Because the dataset consists exclusively of fully anonymized retrospective clinical records with no patient identifiers, and no new patient interventions were performed for this study, formal institutional ethics committee review may not be required; however, such review is currently being sought from the relevant institutional authority. The dataset preparation and release followed accepted ethical principles for the retrospective use of de-identified clinical data.

## CRediT Author Statement

**Ali Mustafa Ali Alshaykha:** Conceptualization, Data curation, Investigation, Methodology, Project administration, Resources, Software, Visualization, Writing – original draft, Writing – review & editing. **Qabas A. Hameed:** Formal analysis, Writing – original draft, Writing – review & editing. **Mohammed Basim Omar:** Software, Validation, Writing – review & editing.

## Data Availability

Mendeley DataOrbscan IIz Multimodal Corneal Topography Dataset of Anterior Axial Power Maps with Structured Clinical Parameters for Unsupervised and Self-Supervised Representation Learning (Original data).
